# Premium Intraocular Lens Implantation After Laser Vision Correction: An Updated Narrative Review of Biometry, IOL Selection, Visual Outcomes, and Clinical Management

**DOI:** 10.3390/jcm15176556

**Published:** 2026-08-25

**Authors:** Abdulmohsen Almulhim

**Affiliations:** Department of Ophthalmology, College of Medicine, Jouf University, Sakaka 72388, Saudi Arabia; akalmulhim@ju.edu.sa; Tel.: +966-50-778-8885

**Keywords:** premium intraocular lens, laser vision correction, post-refractive eyes, biometry, IOL power calculation, refractive prediction error, visual outcomes, dysphotopsias

## Abstract

**Background/Objectives**: Premium intraocular lens (IOL) implantation after laser vision correction (LVC) is increasingly common among post-refractive patients seeking cataract and presbyopia management. Therefore, this review aimed to provide a comprehensive updated synthesis of the challenges and outcomes of premium IOL implantation in post-refractive eyes, including visual quality, patient satisfaction, complications, and practical management approaches for patient selection, refractive surprises, and dysphotopsias. **Methods and Search Strategies**: In this narrative review, relevant articles published in English (January 2016–January 2026) were searched for using appropriate keywords and database indexing terms. The databases used in this review were PubMed/MEDLINE, Embase, Web of Science, and Scopus. **Results**: A total of 127 studies were included. Across post-LVC subgroups, including LASIK, PRK, SMILE, RK, and hyperopic ablation, premium IOL implantation provided good distance and intermediate-range vision, significant spectacle independence, and high patient satisfaction. Modern techniques, including total keratometry, posterior corneal evaluation, swept-source optical coherence tomography biometry, current post-refractive equations, multi-method comparison, and selective intraoperative aberrometry, have enhanced refractive targeting, with ±0.50 D of target refraction commonly used as a key accuracy benchmark, though not all outliers have been eliminated. However, several key challenges persist, including accurate corneal power estimation, personalization, and accurate lens position, which predispose patients to refractive surprises and a high frequency of dysphotopsias. Furthermore, there is heterogeneity in identifying visual outcomes and vision quality across studies. **Conclusions**: This review suggests that successful premium IOL implantation in patients after LVC requires individualized planning and structured management protocols. Future studies should standardize quality-of-vision outcomes that compare emerging optics, adjustable technologies, and AI-assisted prediction across post-refractive subgroups.

## 1. Introduction

The intersection of two major advances in the field of ophthalmic surgery, laser vision correction and premium intraocular lens (IOL) technology, has introduced many possibilities and challenges for contemporary cataract and refractive surgeries [1]. To correct myopia, hyperopia, and astigmatism, millions of patients worldwide have undergone corneal refractive surgery using laser in situ keratomileusis (LASIK), photorefractive keratectomy (PRK), small incision lenticule extraction (SMILE), and radial keratotomy (RK) [2]. With this aging population developing cataracts and presbyopia, there is increasing demand for high-quality IOL solutions to restore both distance and near vision without wearing spectacles [1]. Refractive cataract surgery now prioritizes spectacle independence and quality of vision, making minor residual errors more impactful in post-refractive eyes. Expanding premium IOL options raises expectations, demanding high refractive accuracy and visual quality despite altered corneal optics [3,4].

Premium IOLs, such as multifocal, trifocal, extended depth of focus (EDOF), and toric IOLs, as well as accommodating IOLs, may offer spectacle independence at various focal lengths [1,5]. Nonetheless, they require tailored evaluation, calculations, and counseling. The basic challenge is that the corneal architecture is altered: refractive surgery changes the anterior corneal curve but leaves the posterior surface alone, altering the relationship between the anterior and posterior corneal power and resulting in irregular optical zones that may interfere with IOL performance [6,7]. The primary clinical challenges include the following: (1) improper IOL power calculation because of altered keratometry and estimation of the position of the effective lens, (2) a higher risk of refractive surprises and thus, residual refractive errors, (3) possibly higher dysphotopsia caused by a combination of corneal aberrations and multifocal optics, and (4) the inability to predict visual quality outcomes [6,7,8]. Despite these issues, recent improvements in biometric technology, the IOL power calculation formula, and surgical procedures have enhanced outcomes and increased the candidacy of these premium IOLs in this group.

Refractive unpredictability is a general characteristic of post-refractive eyes and is a combination of measurement errors (the imperfect determination of total corneal power, posterior corneal effects, optical zone irregularity, and tear-film instability) and model errors in IOL equations, most notably effective lens position prediction [7,9]. Recent data are favorable to the use of swept-source OCT biometers, direct posterior corneal evaluation, and methods that integrate total keratometry or measured posterior corneal power to reduce prediction error [10,11,12]. Intraoperative aberrometry has been tested as a method for such multifaceted eyes, and articles have compared its refractive targeting with that of available post-refractive formulas, such as Barrett True-K [13].

Since premium IOL results are extremely sensitive to patient-reported quality of vision and dysphotopsia (not refractive accuracy), recent comparative studies in post-refractive cataract patients have focused on satisfaction, photic phenomena, and functional vision across presbyopia-correcting IOL designs, proving the necessity to incorporate the aspect of visual quality into IOL selection and calculation [14,15]. A key rationale for an updated comprehensive overview is that the field has progressed beyond biometry: more recent premium IOL groups, such as non-diffractive EDOF models, and post-surgical adjustable models such as light-adjustable lenses, are being utilized to solve the issue of refractive uncertainty and satisfaction, with recent post-LVC series reporting high shares of eyes within ±0.50 D of the target [15,16,17]. However, existing reviews have mainly focused on biometry and IOL power calculation, with less integrated discussion of newer premium IOL designs, quality-of-vision outcomes, patient satisfaction, dysphotopsia management, and subgroup-specific issues after different types of LVC. Therefore, an updated clinical synthesis linking calculation strategies, IOL selection, outcomes, complications, and postoperative management is needed.

Therefore, this narrative review was conducted to provide a comprehensive updated synthesis of the challenges and outcomes of premium IOL implantation in post-refractive eyes, including biometry and IOL power calculation strategies across refractive surgery types. It also summarizes evidence on visual quality, patient satisfaction, complications, and practical management approaches for patient selection, refractive surprises, and dysphotopsias.

## 2. Methods (Search Strategy, Screening Process, and Evidence Synthesis)

To synthesize the evidence for the present narrative review, a thorough literature search was conducted in PubMed/MEDLINE, Embase, Web of Science, and Scopus using a combination of database indexing terms and keyword searches relating to prior corneal refractive surgery (LASIK, PRK, SMILE, and radial keratotomy); cataract surgery/IOL implantation; and premium IOL categories, such as multifocal, trifocal, extended depth of focus, etc. The present review included articles published from January 2016 to January 2026. The final literature search across PubMed/MEDLINE, Embase, Web of Science, and Scopus was conducted on 28 February 2026. The complete database-specific literature search strategies are provided in Supplementary Table S1. Articles published from January 2020 onward were emphasized to reflect contemporary IOL platforms, biometric technologies, and calculation strategies. This review considered only published English-language literature with a DOI and excluded expert opinions, case reports, conference presentations, and non-peer-reviewed articles. Duplicate records were removed before screening. Titles and abstracts were then screened for relevance, followed by full-text assessment according to the eligibility criteria. The findings and discussion were synthesized and organized according to the themes. During synthesis, the included evidence was interpreted according to clinical relevance, the consistency of findings, study design, refractive surgery subgroup, IOL type, outcome measures, and practical applicability, rather than being presented only as a descriptive citation summary. This narrative approach was chosen to allow a broad and critical synthesis of the available evidence across heterogeneous study designs, IOL technologies, refractive surgery subgroups, and outcome measures, while allowing discussion of key themes, contextual findings, and practical implications. Given this broad clinical scope and heterogeneity, a narrative rather than a systematic review format was considered more appropriate. Selected systematic elements, such as defined study selection, were included to improve transparency and methodological rigor.

**Figure 1:**
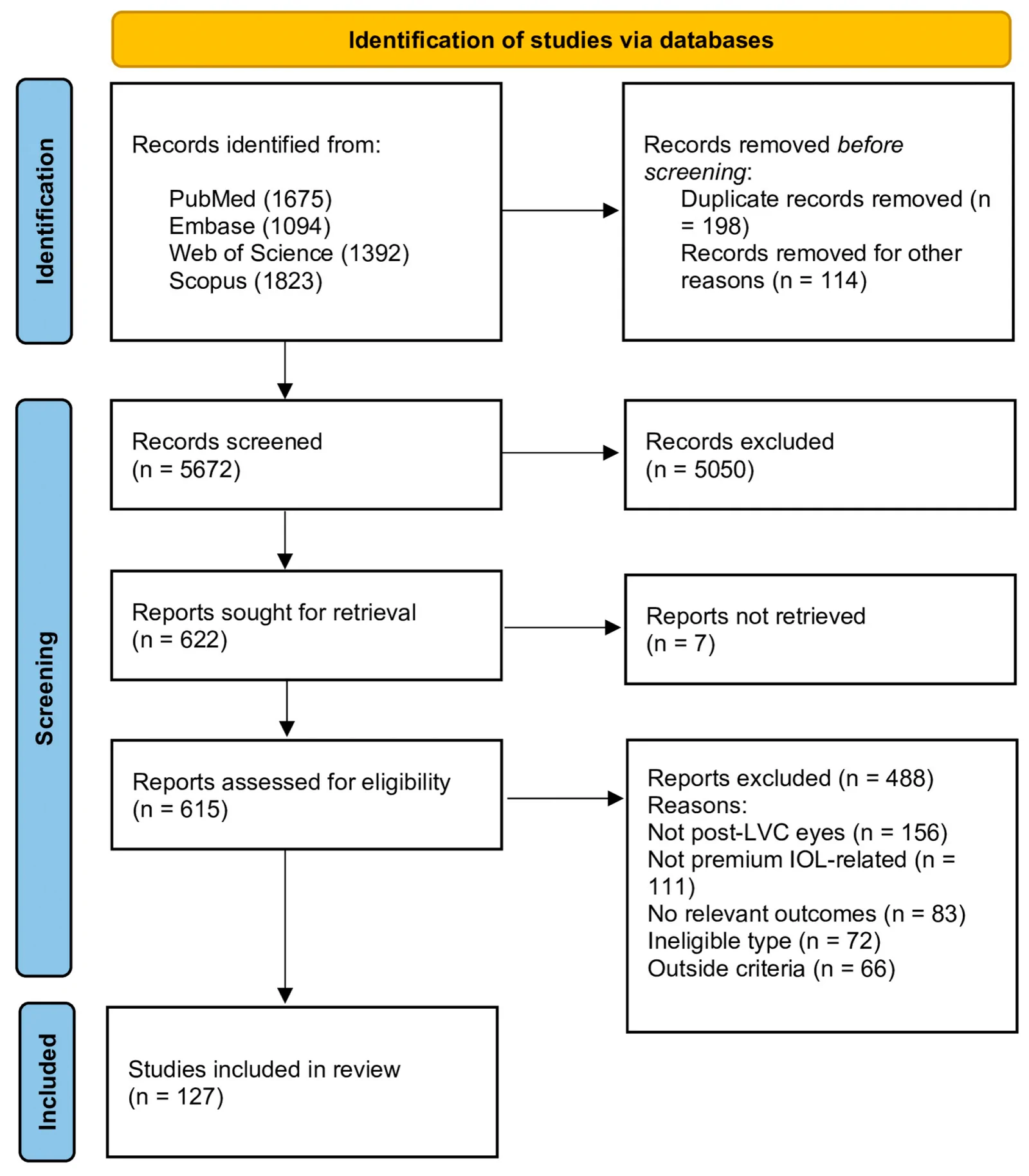
Study selection process.

Two independent experts assisted with retrieving, screening, and thematically arranging the eligible literature, and disagreements regarding inclusion were resolved through consultation with a third expert. This review article includes 127 studies (please see Figure 1 for details of the study selection process). Because this was a narrative review with a broad clinical scope, formal risk-of-bias assessment and evidence grading were not performed.

## 3. Study Background and Theoretical Foundations

### 3.1. Optical Changes Following Refractive Surgery

Corneal refractive surgery essentially alters the eye’s optical properties and has a lasting effect that influences subsequent IOL calculations and visual outcomes throughout the patient’s life. These changes reflect successful premium IOL implantation [18,19]. Besides curvature changes, refractive surgery may alter corneal asphericity, higher-order aberrations (HOAs), and effective optical zone properties, which affect sensitivity and the risk of dysphotopsia following the implantation of presbyopia-correcting IOLs [20,21]. In myopic ablation (LASIK, PRK), the central corneal curvature is flattened, and its refractive power is decreased [18]. This results in a prolate corneal shape with a higher positive spherical aberration compared to the natural oblate corneal geometry [22]. The scale and nature of HOAs can depend on the centration and decentration of the ablation and can be clinically significant when used with diffractive multifocal optics. It alters the anterior–posterior corneal power ratio, rendering the conventional keratometry, which presupposes a constant ratio between anterior and posterior corneal curvatures, no longer valid after ablation. This leads to the systematic overestimation of total corneal power using conventional keratometry, resulting in hyperopic refractive surprises otherwise [8,18]. Ocular surface disease and tear-film instability can also reduce measurement repeatability, are prevalent in patients after refractive surgery, and could be additional contributors to biometric variability and error in predicting refractive measurements [23,24].

Hyperopic ablation surgeries make the cornea of the central eye steeper and more oblate, with greater negative spherical aberration [25,26]. The peripheral cornea is relatively flat, forming a reverse optical zone that can interact unpredictably with multifocal IOL optics. Since the effective optical zone following hyperopic ablation can be smaller and more sensitive to the size of the pupil, patients are able to notice more variation in symptoms in mesopic conditions, which is relevant in counseling and choice of premium IOLs [27,28]. Premium IOL implantation after hyperopic ablation is particularly challenging because corneal power measurements are more variable and refractive prediction errors are more likely [12,18]. Radial keratotomy, which has become less common in recent times, involves radial incisions that flatten the central cornea but cause severe irregular astigmatism and diurnal refractive variation [29,30]. Post-RK eyes are biomechanically unstable and have abnormal optical zones, making them especially difficult candidates for premium IOLs [29,31].

The more recent SMILE procedure also removes an intrastromal lenticule, but not a corneal flap, in theory maintaining more corneal biomechanical integrity than LASIK. Nonetheless, it continues to alter the anterior–posterior corneal power relationship and introduces additional challenges in calculating IOL power, as with other myopic ablation procedures [7,9]. Also, SMILE might generate a variant of the HOA profile for excimer-based procedures, and thus, topography/tomography and aberrometry remain relevant for detecting irregularities that may limit the performance of premium IOLs.

### 3.2. Premium IOL Technologies

There are multiple optical designs classified as premium IOLs, each with benefits and drawbacks for post-refractive surgery eyes.

Multifocal and trifocal IOLs use diffractive or refractive optics to form multiple focal points, allowing the eye to see at distance and near (bifocal) or at distance, intermediate, and near (trifocal) [32]. Diffractive multifocal IOLs separate the incoming light into various diffractive orders, and the energy distribution determines the add power and visual performance at each distance [32]. Trifocal designs have been popular due to their excellent intermediate vision compared to bifocal multifocals [33]. The nature of these lenses, which split light, automatically decreases contrast sensitivity and heightens vulnerability to dysphotopsias, which are further aggravated in post-refractive surgery eyes where corneal aberrations are already present [32,34]. In this regard, corneal regularity and the burden of HOAs are important factors during design selection, particularly before choosing a diffractive multifocal platform in post-refractive eyes [34,35].

EDOF IOLs use a variety of optical principles, such as wavefront shaping, diffractive echelettes, or pinhole optics, to produce the spectrum of functional vision without small focal points [36,37]. Compared to multifocal IOLs, EDOF lenses generally provide higher-quality distance and intermediate vision and fewer dysphotopsias, but near vision can be weaker [37]. They are also tolerant to residual refractive error and produce a low frequency of photic phenomena, which makes them appeal to patients undergoing post-refractive surgery [37,38]. In the EDOF category, non-diffractive wavefront-shaping designs are notable for potentially better preserving contrast and reducing photic effects, which is potentially beneficial in eyes with corneal optics bordering [37,39].

Toric premium IOLs are a combination of astigmatism, multifocal, or EDOF optics. Post-refractive surgery residual or induced astigmatism, especially after RK, is a prominent factor that can severely impair the functionality of presbyopia-correcting IOLs [40]. This problem can be rectified using toric premium IOLs, but precise axis placement and stability are essential. For a post-refractive eye, it is vital to differentiate between regular and irregular astigmatism (and also identify asymmetric or unstable configurations) because toric correction is most efficient in cases when the astigmatism is mainly regular and has the same pattern according to serial measurements [40,41].

Accommodating lenses aim to replicate natural accommodation by altering axial length or shape in response to ciliary muscle contraction [42]. Although theoretically attractive, the accommodative ability of IOLs has demonstrated a less accommodative amplitude in clinical practice and is less frequently used in patients who have undergone previous refractive surgery, where the variability in capsular bag behavior, toleration of residual refractive errors, and predictability of near vision is more often favored by EDOF or other presbyopia-correcting options [42,43].

## 4. Biometry and IOL Power Calculation Challenges

### 4.1. Measurement Prerequisites (Ocular Surface/Topography Stability)

There are numerous sources of systematic error that make accurate IOL power calculation in eyes with prior refractive surgery more difficult. The key issue is that the actual corneal power must be calculated when the anterior surface is surgically altered [7,18]. Previously, several authors have attempted to compare the different formulas [7,44,45]. For example, in their assessment of the visual and refractive outcomes of TFNT IOL implantation (quadrifocal), Choi JY et al. reported that the Barrett True-K formula in the IOLMaster 700 yielded the best refractive outcome [46]. Similarly, a comparative study by Wang et al. found that the Barrett True-K formula could identify IOL power in eyes that had already undergone myopic corneal refractive surgery better than the Haigis-L formula [44]. A recent study (2025) by Ma S et al. that compared 18 IOL formulas stated that the latest formulas, such as Cooke K6, have demonstrated better accuracy [47]. The standard and simulated keratometry of the corneal topography only measure the front surface of the cornea and use a keratometric index that supposes a constant relation between the anterior and posterior curve in the cornea [18,44,48]. This assumption is violated after refractive surgery, leading to the overestimation of corneal power in post-myopic ablation eyes and underestimation in post-hyperopic ablation eyes [7,18,48].

Moreover, tear-film instability and ocular surface disease may compromise the repeatability of measurements, and optimization prior to final biometry is required, especially in post-refractive patients who often have either dry eye or meibomian gland dysfunction [24,49,50,51]. Ablation decentration and irregular astigmatism, as well as small effective optical zones and ectatic changes (seen on tomography), can further cause keratometry distortion, contribute to prediction error, and may also preclude candidacy for diffractive presbyopia correction IOLs [6,52].

Other sources of error include the following: (1) poor prediction of effective lens position (ELP) because formulas that are used in virgin eyes might not predict the effects of altered corneal asphericity and anterior chamber depth relationships post-refractive surgery [7,53]; (2) measurement error because irregular corneal surfaces and optical areas cause measurement variability [24,50]; (3) unavailable reliable historical data on pre-refractive surgery keratometry and post-refractive change [18,54]; and (4) the possibility of finding an altered posterior corneal power that can be caused by certain refractive operations [7,55]. Another factor is incompatible measurements of corneal power zone and the functional visual axis (angle kappa/alpha and pupil size dependence), which may be of relevance when choosing and centering premium optics [56,57].

### 4.2. IOL Power Calculation Methods and Formulas

Various strategies have been formulated to address IOL power calculation issues in post-refractive surgery eyes. The most widely used strategies are as follows:

Historical methods refer to the use of pre-refractive surgery keratometry and the magnitude of the refractive change to compute the true corneal power. These approaches are usually unfeasible, as many patients do not have complete records, though they are valid when reliable historical information is available [18,58]. No-history methods have evolved to compute IOL power without preoperative data. These include the Haigis-L formula, the Barrett True-K formula, the Shammas-PL formula, and a number of regression-based methods [58,59,60]. The Barrett True-K formula has been shown to be much more accurate in various studies and is generally recommended for post-refractive surgery eyes [54,59,60].

The ASCRS post-refractive IOL calculator is also a structured means of reducing outliers and comparing results across various validated systems in clinical practice, even when historical data may be incomplete [60,61]. OCT-based biometry anterior corneal power measurements alone would cause less error compared to posterior corneal curvature and total power measurements collected using swept-source optical coherence tomography (SS-OCT) devices [8,62]. Previous research has demonstrated that SS-OCT-based formulae can produce errors of 0.50 or less for the majority of post-refractive surgery eyes [63]. In addition to SS-OCT, ray-tracing methods, which assume posterior and anterior corneal surfaces, are widely known for customizing corneal power estimates, especially in eyes with non-standard corneal profiles or increased aberrations [64,65]. Aberrometry is a procedure performed during surgery after IOL implantation to assess the eye’s status, enabling real-time adjustment of IOL power. This technology has demonstrated potential in post-LASIK eyes, with studies reporting better refractive results than those achieved by formula-based calculations before the operation. Studies comparing intraoperative aberrometry with preoperative post-refractive formulas have reported mixed findings. For example, Dawson VJ et al. [66] did not find any significant difference between intraoperative aberrometry and the Barrett True-K formula, while Gouvea L et al. found positive results in favor of intraoperative aberrometry [67]. Other studies have also reported favorable refractive predictability with intraoperative aberrometry in selected post-refractive eyes [68,69]. Taken together, these findings suggest that intraoperative aberrometry may be useful in selected cases, but its performance can be affected by ocular surface quality, corneal edema, and viscoelastic use; therefore, it should be viewed as a complement to, rather than a substitute for, careful preoperative planning [66,68,69].

Multiple formula averaging involves calculating IOL power using multiple formulas and averaging the results, with the aim of minimizing the likelihood of outliers and improving overall accuracy. Research findings indicate that a significant proportion of eyes achieve an average target refraction of ±0.50 D when using multi-formula averaging techniques [59,60]. In cases where formula yields vary significantly, it can be useful to first give preference to techniques that account for posterior corneal power/total keratometry and ensure consistency between repeat measurements before finalizing IOL power [63,70,71].

Overall, the evidence suggests that no single calculation strategy is consistently superior across all post-LVC eyes; therefore, formula agreement, measurement quality, posterior corneal assessment, and clinical judgment should be considered together when selecting IOL power.

### 4.3. Prediction Error and Refractive Accuracy

Although improvements in calculation techniques have reduced refractive prediction errors, they are as good as or more pronounced in post-refractive surgery eyes than in virgin eyes [21,72,73]. The nature and extent of the prediction error depend on the type of prior refractive surgery, the method used to calculate the IOL, and individual patient considerations [21,48]. Since the tolerance of the premium IOL to residual sphere and cylinder is minimal, reporting of both spherical equivalent accuracy and residual astigmatism (and their correlation with patient-reported symptoms) is especially important when considering cohorts of post-refractive patients [38,74].

A number of studies have identified factors influencing prediction error in post-refractive surgery using premium IOLs. Yoo et al. established that the type of multifocal IOL, IOL power calculation formula, and the strength of previous refractive correction had an impact on prediction error [48]. Corneas with greater levels of previous myopic correction were more likely to have bigger prediction errors, probably because they have more modifications in corneal structure. Target refraction is an important selection. Although small amounts of myopia can be tolerated using monofocal IOLs, premium IOLs need a high level of emmetropia to maximize the multifocal or EDOF effect. Refractive errors outside this range may have a profound negative effect on visual outcomes and patient satisfaction [21,48,74].

In addition, the residual refractive error management plan (spectacles, corneal enhancement, piggyback IOL, or IOL exchange) must be addressed preoperatively and during counseling, as the trend of intervention is usually lower in premium IOL recipients [75,76].

## 5. Premium IOL Types and Clinical Applications

### 5.1. Multifocal/Trifocal

Multifocal and trifocal IOLs represent the most extensively studied premium IOL category in post-refractive surgery eyes [21,77]. It has been shown that these lenses can produce functional vision at a variety of distances, though results vary depending on the type of IOL, the type of refractive surgery performed before it, and refractive accuracy [78,79]. Diffractive multifocal IOLs have been successfully implanted in post-LASIK and post-PRK eyes, with mixed results [78,79]. Chang et al. stated that myopic LASIK patients with diffractive multifocal IOLs had good visual acuity and quality of life, with significant variation in IOL formula accuracy [79]. The paper highlighted the need for adequate power calculation to achieve the best results with these lenses.

The use of trifocal IOLs has become increasingly common because their intermediate vision is superior to that of bifocal designs. A series of studies has been conducted to investigate trifocal IOL outcomes in post-refractive surgery eyes [21,52,77]. Li et al. have noted that the implantation of trifocal diffractive IOLs (AT LISA tri 839 MP) following myopic LASIK or PRK resulted in mean monocular uncorrected visual acuities of 0.02 ± 0.07 logMAR at distance, 0.10 ± 0.10 logMAR at intermediate distance, and 0.15 ± 0.11 logMAR at near distance. Spectacle independence occurred in 81% of the patients, and 94% reported satisfaction scores ≥ 3.5 of 4.0 [52]. Blaylock JF and Hall BJ investigated the refractive results of using trifocal IOLs in post-myopic LASIK and PRK eyes, as they discovered that patient selection and proper IOL power calculation were key factors to success. The research indicated that patients’ expectations regarding refining possible residual refractive error should be managed [80]. Trifocal IOL models in post-refractive surgery eyes have been compared, and performance differences have been identified as being dependent on the profile of the sphere aberration [19,77].

Mayordomo-Cerda et al. compared two trifocal IOL models on eyes that had undergone hyperopic corneal ablation: PhysIOL FineVision Pod-F (negative spherical aberration) and Rayner RayOne Trifocal (neutral spherical aberration). Although there was no significant difference in visual acuity outcomes among the groups, the FineVision Pod-F showed a much higher predictability of refraction (*p* < 0.001), and both lenses had similar levels of patient satisfaction [77]. This implies that the IOL spherical aberration profile can interact with post-ablation corneal aberrations, affecting refractive outcomes. Secondary trifocal IOL implantation has been discussed as a presbyopia-correcting method in patients who had previously been fitted with monofocal IOLs following refractive surgery. However, in secondary trifocal- or sulcus-based implantation, refractive outcome may also be influenced by the IOL platform itself, because differences in haptic design, material stiffness, and possible anterior movement can affect the effective lens position and final refractive result [9,81]. A secondary trifocal sulcus IOL implantation study on patients who had undergone myopic laser vision surgery and cataract surgery showed excellent distance and near vision with a binocular uncorrected distance visual acuity of 0.1 logMAR (or above) in all subjects and a binocular uncorrected near visual acuity of 0.1 logMAR (or above) in all except one subject. This method is an option that could improve near and intermediate vision for appropriately selected patients [81].

### 5.2. EDOF/Wavefront-Shaping/Enhanced Monofocal

EDOF IOLs have emerged as an attractive alternative to multifocal IOLs in post-refractive surgery due to their reduced dysphotopsia profile and greater tolerance to residual refractive error [38,39,82,83]. Non-diffractive EDOF IOLs have demonstrated promising results in demanding post-refractive surgery cases [15]. Researchers have concluded that EDOF IOLs have the potential to provide functional vision across a range of refractive errors while supporting functional vision in complex eyes [38,84]. One study specifically designed to determine EDOF IOL tolerance to refractive errors in patients who had undergone prior corneal refractive surgery concluded that the lenses had a safety margin, which is especially important in this group [38].

Comparative studies have also been conducted on EDOF IOL performance in comparison to multifocal and monofocal IOLs in post-LASIK eyes. Bucci reported patient satisfaction, visual results, and regression analysis in patients who received multifocal, EDOF, or monofocal IOL implants after LASIK [85]. The research studies concluded that although multifocal IOLs provided the best near vision, EDOF IOLs offered a better trade-off between distance and intermediate vision, with lower incidences of dysphotopsia and, therefore, were ideal for patients who needed moderate near vision after refractive surgery [82,83,85]. Extended range of vision IOLs are a further development in EDOF technology. The clinical outcome was tested in Carreras et al., who found good distance and intermediate vision, and satisfactory near vision, with an extended range of vision IOL following LASIK, with minimal associated photic phenomena [86]. The paper highlighted that these lenses might be especially suitable for post-LASIK patients, who do not prioritize maximum near vision performance as much as distance and intermediate vision. Lin et al. conducted a direct comparative study of the impact of prior LASIK on the calibration accuracy of EDOF IOLs. The study established that whereas previous LASIK had some implications for IOL performance, EDOF lenses provided functional vision at most distances and thus their application in this population could be considered. Furthermore, they suggested that a longitudinal follow-up study is necessary to confirm their results [3].

Taken together, EDOF and enhanced monofocal designs appear clinically attractive in post-LVC eyes because they may offer a better balance between functional range and tolerance to residual refractive error; however, the strength of evidence remains limited by heterogeneous IOL platforms, small cohorts, and limited direct comparisons.

### 5.3. Toric Premium IOLs

Eyes that have undergone refractive surgery have residual or induced astigmatism, which can greatly compromise the performance of presbyopia-correcting IOLs [21,87]. Toric premium IOLs solve this problem by correcting astigmatism with EDOF or similar optics [88,89]. For example, in 2024, Ruiz-Mesa R et al. demonstrated that toric pure refractive EDOF technology IOLs provide good visual outcomes and high patient satisfaction [88]. Astigmatism management is particularly important in eyes that have undergone RK surgery, as these eyes often exhibit irregular astigmatism and biomechanical instability [90]. Research has demonstrated that in such difficult cases, proper evaluation of corneal astigmatism and application of toric IOLs can help improve outcomes [91,92]. Toric premium IOLs have been used successfully to treat both presbyopia and astigmatism in post-hyperopic ablation eyes, for which optical problems are unique due to corneal steepening [89,93]. The measurement of total corneal astigmatism, including posterior corneal astigmatism that may be altered by refractive surgery, is key to success [12,71,91].

### 5.4. Accommodating/Novel Designs

Rotationally asymmetric refractive multifocal IOLs are a new technology that can potentially bring benefits to post-refractive surgery eyes through presbyopia correction [94]. Results of rotationally asymmetric refractive low-addition multifocal IOL implantation have been reported in patients who had undergone prior hyperopic corneal laser refractive surgery (Zhang et al., 2025) [95]. Similarly, Nouzovska et al. studied these lenses in patients who had undergone corneal laser myopic refractive surgery [96]. The designs are intended to correct presbyopia with minimum dysphotopsia due to their special optical design [94,96].

Light-adjustable IOLs and other new technologies allow refractive result adjustment after implantation, which might be especially useful in post-refractive surgery eyes, where prediction errors are more frequent [4,17,97]. In their article, Susanna et al. addressed the use of light-adjustable IOLs as one of the overall principles of success in cataract surgery after refractive surgery [4].

Small-aperture IOLs use pinhole optics to lengthen the depth of focus and have been suggested as an alternative in post-refractive surgery eyes, especially those with irregular corneal optics [4,36]. Although they are not as prevalent in this population, they are another option to consider for a chosen patient [36,98].

## 6. Visual Outcomes

### 6.1. Post-LASIK Eyes

LASIK is the most common refractive surgery procedure, and consequently, the majority of evidence on premium IOL outcomes in post-refractive surgery eyes comes from post-LASIK populations.

Post-LASIK eyes with premium IOLs typically achieve good visual acuity when precise IOL power calculation is achieved [21]. Various studies have reported uncorrected distance visual acuity (UDVA) results of 0.10 logMAR or higher in most eyes with good patient satisfaction [52,80,85,99]. Intermediate and near vision also differ depending on the type of IOL, with trifocal IOLs typically providing better near vision than EDOF lenses [100]. Fisher et al. compared the clinical results between distance-dominant multifocal and monofocal IOLs when used in post-LASIK cataract surgery scheduled with intraoperative aberrometry [99]. The researchers determined that intraoperative aberrometry has the potential to increase refractive accuracy and that distance-dominant multifocal IOLs provided functional near vision without compromising good distance vision. Modern calculation methods have enhanced refractive predictability in post-LASIK eyes. Research on multiple formula averaging or higher-order formulas, such as Barrett True-K, indicates that a sizable proportion of eyes can achieve a target refraction within ±0.50 D [60,101]. Nonetheless, this is still lower than the predictability normally attained in virgin eyes.

### 6.2. Post-PRK Eyes

PRK produces optical modifications equivalent to those of LASIK without a corneal flap, which may have more stable biomechanics [102,103]. Results for premium IOLs in post-PRK eyes tend to be similar to those in post-LASIK eyes. Li et al. included post-LASIK and post-PRK eyes in their investigation of trifocal IOL implantation, with no significant differences in visual outcomes between the two groups [52]. This means that the type of myopic ablation procedure (LASIK vs. PRK) is not as significant as the extent of refractive correction and precision of IOL power calculation. Similar results were reported by Blaylock JF and Hall BJ, who compared refractive outcomes after trifocal IOL insertion in post-myopic LASIK eyes and PRK eyes, finding no significant difference between the groups [80]. The study highlights that both processes pose comparable issues for IOL power computation and that the management principles are equivalent.

### 6.3. Post-SMILE Eyes

SMILE is a more recent type of refractive surgery, which eliminates an intrastromal lenticule and uses no corneal flap. Although it theoretically offers benefits for corneal biomechanical stability, SMILE poses the same IOL power calculation problem as LASIK and PRK. Limited information is available on the results of premium IOLs in eyes that have undergone SMILE because this procedure has become popular only in recent years. The optical principles and calculation issues, however, are no different from those of other myopic ablation procedures, and parallel management approaches can most probably be used.

### 6.4. Post-Radial Keratotomy Eyes

RK poses unique problems for high-quality IOL implantation due to irregular astigmatism, biomechanical instability, and diurnal refractometry. Despite these problems, several studies have also shown that premium IOLs can be effectively implanted in select post-RK patients.

In a study (2024) by Aljindan et al., visual performance and patient satisfaction following the implantation of a trifocal IOL in cases of radial keratotomy were reported [104]. The researchers concluded that, with carefully selected post-RK patients, it was possible to achieve functional vision at various distances, with results worse than those of post-LASIK eyes [104]. Martin-Escuer et al. studied refractive correction with multifocal IOLs following radial keratotomy and found that, although there are certain issues, good results can be achieved when patients are selected appropriately [105]. The paper highlighted the importance of evaluating corneal stability and irregular astigmatism prior to the implantation of a premium IOL.

Agarwal et al. reported spectacle independence in patients with prior radial keratotomy after cataract surgery and demonstrated that, with appropriate management, including optimal IOL selection and realistic patient expectations, good outcomes are possible [106].

### 6.5. Post-Hyperopic Ablation Eyes

Hyperopic ablation procedures produce different optical problems than myopic ablation, which include steepening of the central cornea, an oblate cornea, and more variation in corneal power measures [27,107,108]. These factors may affect refractive predictability and visual outcomes after premium IOL implantation in post-hyperopic ablation eyes.

In their study, Mayordomo-Cerda et al. specifically investigated outcomes of trifocal IOLs in eyes with a history of corneal ablation for hyperopia correction [77]. The study confirmed that, even though the overall visual acuity results were good, refractive predictability varied more than in post-myopic ablation eyes, and the IOL models showed significant differences in prediction error. This underscores the importance of IOL choice and calculation in post-hyperopic ablation eyes. Zhang et al. studied rotationally asymmetric refractive low-addition multifocal IOL implantation in patients who had undergone hyperopic corneal laser refractive surgery and investigated whether other IOL designs could benefit this difficult population [95].

The research proposed that new IOL designs could address specific optical problems in post-hyperopic eyes following ablation [27,95]. Brenner et al. studied presbyopic refractive lens exchange with trifocal IOL surgery following corneal laser vision correction in both myopic and hyperopic cases [109]. The experiment involved a thorough biometry analysis and found that various averaging formulas improved refractive accuracy in both myopic and hyperopic ablation groups.

## 7. Patient Satisfaction and Quality of Vision

With appropriate patient selection and surgical planning, patient satisfaction with premium IOLs in post-refractive surgery eyes is usually high, although slightly lower than virgin eyes because of higher rates of dysphotopsia and refractive surprises [14,21,110]. Most notably, dissatisfaction in this setting is often multidimensional, and postoperative assessment must be carefully considered to help differentiate lens optic symptoms from treatable ocular surface disease and posterior capsule opacification conditions [4,111,112].

One of the main objectives of the premium IOL implantation process is spectacle independence, which is a key driver of patient satisfaction [21,52,79]. Focusing on eyes that have undergone laser vision correction, a meta-analysis by Sun et al. showed that spectacle independence rates were 98%, 99%, and 78% for near, intermediate, and distance vision, respectively, with presbyopia-correcting IOLs [21]. These are indeed comparable to the rates achieved in virgin eyes, implying that, if refractive accuracy is met, premium IOLs can effectively restore functional vision at all distances [17,21].

In some studies, quality-of-life outcomes have been measured. According to Chang et al., diffractive multifocal IOL implantation following myopic LASIK resulted in good quality-of-life scores, and patients reported being satisfied with their vision for day-to-day tasks [79]. Li et al. reported a 95.00 ± 7.29 average composite score of 100 on the VF-14 questionnaire, which indicates high visual functioning-related quality of life [52]. In the literature, patient-reported outcome measures often include both functional and symptom burden vision, with a focus on quality of vision (contrast and photic phenomena) as significant as acuity to perceived success [14,21,110].

Patient satisfaction scores vary in the literature but are mostly positive. Li et al. found that 94 percent of patients rated their satisfaction ≥ 3.5 out of 4.0 after trifocal IOL implantation after myopic laser correction [52]. The authors observed that two trifocal IOL models were equally satisfactory for patients in post-hyperopic ablation cases, although they differed in refractive predictability [77]. This indicates that patients can tolerate small refractive errors provided that visual function is normal and expectations are properly controlled. Nevertheless, even minor residual cylinder or decentration-related symptoms may be poorly tolerated by members of some groups, especially those with high night-driving requirements [110,113].

The factors influencing satisfaction include refractive accuracy and the level of dysphotopsia, preoperative counseling, and patient characteristics [21,110,114]. It is well established that patients who obtain their desired refraction and have realistic expectations of the possible visual effects are more satisfied. In a number of studies, comparative satisfaction with IOL types has been discussed. Bucci found that multifocal IOLs were most effective for near vision, but overall satisfaction was similar because EDOF IOLs had lower dysphotopsia rates and higher intermediate vision (high satisfaction level) [85]. This underscores the need to align IOL choice with the visual requirements and tolerance of optical phenomena of the individual patient. For this purpose, non-diffractive extended range/EDOF designs can be considered for patients with borderline corneal optics or reduced tolerance to photic symptoms, and diffractive multifocal/trifocal designs can be best suited for highly motivated patients who value near spectacle independence [4,21,114].

Ni et al. also compared the visual and patient satisfaction outcomes of three presbyopia-correcting IOL types in eyes that had undergone corneal refractive surgery, and revealed the performance of various IOL technologies in this cohort. The paper underlined that various IOL devices can achieve satisfactory results, and the choice must be personalized based on patient-specific factors and the surgeon’s experience [14]. Key factors in satisfaction include achieving the target refraction and matching IOL choice to patient needs. Park (2026) emphasized managing dry eye, corneal aberrations, and expectations to maximize satisfaction [115]. In 2022, Dołowiec-Kwapisz A et al. reported that a non-diffractive EDOF-IOL achieved better postoperative satisfaction while avoiding adverse events associated with multifocal lenses [116]. Therefore, satisfaction outcomes should be interpreted cautiously, because studies vary in follow-up duration, IOL design, prior LVC type, refractive accuracy, and the instruments used to measure quality of vision and dysphotopsia.

## 8. Complications and Adverse Events (Optical vs. Surgical)

While premium IOLs can achieve excellent outcomes in post-refractive surgery eyes, several complications and adverse events occur at higher rates than in virgin eyes.

### 8.1. Optical Complications (Dysphotopsia and Functional Symptoms)

The most frequent complaint in post-refractive surgery eyes is dysphotopsia following premium IOL implantation. A study by Sun et al. established that 40% of patients (95% confidence interval, 0.25–0.64) had halos and 31% (95% confidence interval, 0.16–0.60) had glare [21]. The rates are higher than expected in virgin eyes, and this is probably due to the combination of corneal aberrations following previous refractive surgery with the diffractive or refractive optics of high-end IOLs [21,117].

Li et al. also reported that 19% of patients had halos and glare 3 months after trifocal IOL implantation following myopic laser correction [52]. Notably, dysphotopsias in the majority of cases can improve with neuroadaptation over time; however, some individuals may require intervention (Alio JL et al.) [117]. Night-driving difficulty is a common functional problem in symptomatic individuals, which is most often influenced by pupil size, residual refractive error (particularly residual astigmatism), and existing corneal HOAs [114,118].

### 8.2. Refractive Surprises and Residual Astigmatism Impact

A higher rate of refractive surprises is observed in eyes that have undergone post-refractive surgery due to the difficulty in calculating IOL power. Even though studies indicate that a good proportion of eyes have within ±0.50 D of the target refraction, it is still less than the >90% of virgin eyes reported [80,119,120]. Even high-end IOLs have a significant risk of refractive errors that may necessitate the use of spectacles, contact lenses, or refractive-enhancing procedures [119,121]. Even at low levels of residual cylinder, disproportionate decreases in satisfaction with toric and presbyopia-correcting optics indicate that astigmatism planning and verification are critical [118].

### 8.3. Posterior Capsule Opacification and Optical Degradation

The incidence of posterior capsule opacification (PCO) in post-refractive surgery eyes is the same as in virgin eyes, and Nd: YAG laser capsulotomy can be used to successfully treat this condition. However, PCO can be more significant for visual quality in eyes with premium IOLs, since multifocal and EDOF optics are more sensitive to optical degradation [122,123]. Before attributing symptoms to the IOL design, it is necessary to rule out PCO or subtle decentration/tilt in a clinical setting, especially in patients who are not satisfied with their premium IOL implant [114,124].

### 8.4. Other Complications

Other complications found in the literature are rare cases of IOL decentration, which may be observed with multifocal and toric IOLs, and rare cases of cystoid macular edema or other postoperative inflammation [124,125]. Occasionally, IOL exchange may be needed when refractive surprises are large, or patients cannot tolerate dysphotopsias. Although it is not widely reported in the literature, IOL exchange is a crucial management option for severe complications. Since exchange becomes more difficult over time, early identification of ongoing severe symptoms and timely referral should be considered when first-line interventions are ineffective [112].

Beyond the complications already discussed, post-LVC patients receiving premium IOLs have some distinct “quality-of-vision” adverse patterns reported in the literature. Even when Snellen acuity is good, contrast sensitivity and mesopic performance can be reduced, and subjective vision quality may remain limited in a subset of post-LASIK/PRK premium IOL recipients and may require enhancement [79,126]. Another complication that can lead to dissatisfaction that should be handled appropriately is neuroadaptation failure. Wavefront-guided LASIK has been reported by Seiler TG et al. as a targeted rescue strategy to improve satisfaction and spectacle independence [127]. In addition, post-LVC corneas with higher baseline spherical aberrations/HOAs may be more vulnerable to “IOL–cornea interaction” effects, so some premium IOL series in post-myopic LASIK/PRK specifically highlight the role of corneal aberration profile in counseling and outcomes, even when refraction is close to the target [15,128].

Binocular vision should also be considered as a possible non-IOL source of postoperative dissatisfaction. In post-LVC patients, especially those with previous monovision, anisometropia, ocular dominance mismatch, reduced stereopsis, or decompensated phoria/strabismus, good monocular visual acuity may coexist with asthenopia, diplopia, impaired depth perception, or poor adaptation to presbyopia-correcting optics [35,114,129]. Therefore, persistent postoperative complaints should prompt assessment of binocular function and interocular refractive balance before symptoms are attributed solely to premium IOL. The vitreous body is another potential contributor, particularly in previously myopic eyes. Vitreous opacities, posterior vitreous detachment, vitreoretinal traction, retinal breaks, or lattice degeneration may cause floaters, photopsia, reduced subjective visual quality, or persistent visual disturbance after cataract/IOL surgery. Therefore, dilated posterior-segment examination and OCT or fundus assessment should be considered when symptoms are disproportionate to anterior-segment, refractive, or IOL-related findings [130,131].

## 9. Management Strategies and Clinical Recommendations

### 9.1. Preoperative Assessment and Patient Selection

The following clinical recommendations should be interpreted as practical considerations derived from the narrative synthesis rather than formally graded evidence-based recommendations.

Effective premium IOL implantation in post-refractive surgery eyes is initiated by thorough pre-assessment and proper patient selection before surgery. Some authors discussed what makes post-LVC cataract surgery difficult, and the need to conduct an elaborate preoperative examination is paramount [132,133]. Supplementary Table S2 summarizes a practical decision-making framework derived from the included literature and the clinical themes discussed in this review.

Important preoperative data involve the following: (1) a detailed history of refractive surgery, including the type of procedure, date, preoperative refraction, and amount of correction; (2) current refraction and visual acuity; (3) corneal topography and tomography to evaluate regularity and optical zone; (4) multiple keratometry measurements with various instruments; (5) anterior-segment OCT to measure corneal thickness and the anterior chamber; (6) optical biometry with multiple measurement instruments where possible; and (7) ocular surface health and tear-film evaluation [6,9,129].

Patient selection should take into account the following: (1) realistic expectations and awareness of possible dysphotopsia and refractive surprises; (2) stable refraction at least 1 year after refractive surgery; (3) regular corneal topography with no substantial irregular astigmatism; (4) sufficient optical zone size with thresholds considered as practical guidance rather than absolute cut-offs (normally greater than 5.5 mm in the case of multifocal IOLs); (5) no significant ocular surface disease or dry eye; and (6) no other ocular pathology that may lead to limitations of visual potential [6,73,129].

Factors that may limit candidacy for premium IOL implantation include the following: (1) irregular corneal topography with high higher-order aberrations; (2) unstable refraction or progressive hyperopic shift (especially in post-RK eyes); (3) severe dry eye disease; (4) unrealistic patient expectations or intolerance to any possible dysphotopsia; and (5) very small optical zones (less than 5 mm), with this threshold interpreted as a practical consideration rather than an absolute contraindication [4,9,116,129].

### 9.2. IOL Selection Strategies

IOL selection plays a very important role in improving outcomes after refractive surgery.

Matching IOL to patient needs: It is important to take into consideration the following: (1) visual requirements and lifestyle (EDOF may be preferable to high-add multifocal); (2) tolerance to dysphotopsias (lower tolerance is better with EDOF or monofocal); (3) the amount of prior refractive correction (greater corrections may favor consideration of EDOF); (4) corneal aberration profile (greater aberrations may favor consideration of EDOF); and (5) astigmatism (toric IOL may be considered) [4,15,19].

Regarding IOL design, the profile of spherical aberration should be used in a manner that is complementary to post-ablation corneal aberrations [4,134]. Mayordomo-Cerda et al. observed variations in refractive predictability with trifocal IOLs featuring negative and neutral spherical aberration in eyes undergoing post-hyperopic ablation therapy, postulating that aberration profile matching may affect outcomes [77].

### 9.3. Refractive Surprise Management

Refractive surprises are still more prevalent in post-refractive surgery eyes and may benefit from a systematic management approach, despite the best calculation techniques [4].

Within the range of mild refractive errors (−0.50 to ±1.00 D), conservative treatment may be observed during neuroadaptation, especially with EDOF IOLs that are more tolerant of residual error. Some patients who have working binocular vision often adapt well to mild myopia or hyperopia. Moderate to large refractive errors (>±1.00 D) are usually something that may warrant intervention depending on symptoms and patient expectations. They can be corrected by the following: (1) spectacle correction to particular tasks, (2) contact lens correction, (3) laser corneal enhancement (LASIK, PRK, or SMILE), (4) add-on/piggyback IOL implantation in selected anatomically suitable cases, or (5) IOL exchange in severe cases [9,15,21,73,112].

In post-refractive surgery eyes, laser enhancement after premium IOL implantation has been reported to yield generally positive results. A study on laser corneal enhancement following trifocal IOL implantation in eyes with a history of photo-ablative corneal refractive surgery concluded that enhancement procedures could correct residual refractive error and improve patient satisfaction. Among the key considerations are sufficient corneal thickness, stable refraction, and patient expectations [21,121,127]. Determining the intervention timeline is also important. The majority of surgeons suggest allowing 3–6 months after IOL implantation for refractive stabilization and neuroadaptation before considering enhancement procedures [4,127].

Depending on patient objectives, residual astigmatism in patients with toric premium IOLs may be evaluated using axis alignment and rotational stability in the early stages and by corneal enhancement or optical correction in the late stages, as the astigmatism remains stable and the cornea is suitable [4,9].

### 9.4. Dysphotopsia Management

Premium IOL implantation in post-refractive surgery eyes is associated with dysphotopsias that require appropriate clinical management. An important preventive measure is preoperative counseling. The probability of halos, glare, and starbursts, and the possibility of neuroadaptation with time ought to be elucidated to patients in detail. Reasonable expectations minimize the chances of disappointment [21,80,127].

Most may initially be managed conservatively while neuroadaptation occurs after 3–6 months. The strategies are as follows: (1) reassurance and education, (2) ocular surface health optimization, (3) pupil-constricting drops in rare cases, and (4) outdoor glare-tinted lenses. In severe cases that do not improve over time, surgical intervention may be considered. It can be combined with an IOL exchange with either a monofocal or EDOF lens with a lower dysphotopsia profile, a piggyback IOL to alter optical characteristics, or, in rare cases, IOL explantation. Surgical intervention should be decided with care, as outcomes cannot always be predicted [73,112].

For dysphotopsias, ocular surface disease, residual refractive error (including small amounts of astigmatism), and IOL position or tilt should generally be reassessed and optimized before surgical intervention is resorted to, because these factors can make a meaningful difference for premium IOL patient symptoms [21,52]. Because high-quality comparative evidence on postoperative management pathways remains limited, management decisions should be individualized and should first address reversible causes before considering surgical intervention.

## 10. Knowledge Gaps and Future Directions

The field of premium IOL implantation in post-refractive surgery eyes is evolving rapidly, but several important knowledge gaps have been identified in the present review as follows:

- The absence of prospective, multicenter investigations paying specific attention to premium IOLs in post-refractive eyes.
- Very high heterogeneity in describing the details of previous refractive procedures (LASIK/PRK/SMILE/RK/hyperopic), ablation magnitude, optical zone size, and post-surgical time.
- Inconsistent measurement and reporting of posterior corneal power, total keratometry, and corneal higher-order aberrations, limiting comparison across studies.
- Limited head-to-head comparisons of the current categories of premium IOLs used in post-refractive eyes (diffractive trifocal/non-diffractive EDOF/wavefront-shaping/enhanced monofocal/adjustable lenses).
- Low-risk and high-risk subgroups, such as patients with a post-RK history and hyperopic ablations, high myopic corrections, and irregular corneas, were underrepresented, and little data was available on long-term stability.
- There are insufficient standardized tools for patient-reported outcomes or dysphotopsia; satisfaction is often reported using insufficiently validated quality-of-vision tools.
- Evidence-based candidacy criteria (acceptable HOA load, topography/tomography red flags, pupil size, and ocular surface stability requirements) are still unclear.
- Inadequate comparative evidence on management pathways for dissatisfaction (when and which type to implement between improvement, piggyback IOL, realignment, or IOL exchange).
- In post-LVC eyes, the cost-effectiveness and practical use of adjunct technologies (intraoperative aberrometry, image-guided alignment) are questionable.
- Most available studies are retrospective or observational, with heterogeneous populations, IOL designs, follow-up periods, and outcome measures; this limits direct comparison and reduces the certainty of clinical recommendations.

Considering the existing knowledge gaps, several future directions are likely to shape clinical practice and research priorities related to premium IOLs:

- The integration of advanced biometrics with the use of total keratometry, direct anterior measurements of the cornea, and better quality images to minimize refractive prediction error.
- IOL power prediction models supported by artificial intelligence and machine learning and trained on high amounts of post-refractive data to enhance accuracy and decrease outliers.
- Increased application and testing of light-adjustable and other adjustable IOL technologies to overcome post-refractive unpredictability without corneal enhancement.
- Next-generation design of high-end IOLs (enhanced EDOF, hybrid EDOF-multifocal, non-diffractive wavefront-shaping, and contrast-preserving optics) specifically in post-refractive cornea.
- More advanced guided IOL choice systems that include corneal aberration mapping, optical zone measurements, pupil behavior, lifestyle requirements, and symptom acceptance.
- Obtaining results for post-refractive premium IOLs on residual astigmatism, functional vision parameters, validated dysphotopsia scoring, and patient-reported quality-of-vision instruments.
- Long-term prospective research on satisfaction persistence, postoperative dysphotopsia, PCO effects, the centration of IOLs, and age-related eye comorbidities.
- Comparative effectiveness trials to establish optimal strategies in each subgroup (post-RK vs. post-LASIK/PRK/SMILE; hyperopic vs. myopic ablations; regular vs. borderline corneas).
- A methodological limitation of this narrative review is that formal risk-of-bias assessment, certainty-of-evidence grading, and quantitative meta-analysis were not performed because of the broad clinical scope and heterogeneity of included study designs, IOL technologies, refractive surgery subgroups, and outcome measures. Therefore, the clinical recommendations presented should be interpreted cautiously as considerations derived from the narrative synthesis rather than formally graded evidence-based recommendations.

## 11. Conclusions

Premium IOL implantation after laser vision correction is increasingly successful and can achieve high levels of functional vision and patient satisfaction when patients are carefully selected and counseled. Nonetheless, the results are not as predictable as in virgin eyes due to the effects of corneal optics that complicate corneal power measurement and dysphotopsia prediction. Also, the interplay between existing corneal aberrations and corneal optics that correct presbyopia may enhance the likelihood of refractive surprises. The best outcomes depend on careful preoperative assessment, advanced post-refractive calculation methods, individualized IOL selection, and structured postoperative complaint management. Further improvements in refractive accuracy and quality of vision might be achieved by continuing to develop biometry, adjustable IOL technologies, and AI-assisted prediction models. Moreover, for successful premium IOL implantation among patients after LVC, individualized planning and structured management protocols are necessary. Future studies should standardize quality-of-vision outcomes that compare emerging optics, adjustable technologies, and AI-assisted prediction across post-refractive subgroups.

## Data Availability

Not applicable. This is a review article. No new datasets were generated or analyzed during the current study.

## Supplementary Materials

The following supporting information can be downloaded at: https://www.mdpi.com/article/10.3390/jcm15176556/s1, Supplementary Table S1: Complete Database-Specific Literature Search Strategies. Supplementary Table S2: Practical clinical decision-making framework for premium IOL implantation after laser vision correction.
