# Supplementary material for: Premium Intraocular Lens Implantation After Laser Vision Correction: An Updated Narrative Review of Biometry, IOL Selection, Visual Outcomes, and Clinical Management
Source: J Clin Med. 2026 Aug 25;15(17):6556. doi: 10.3390/jcm15176556 (PMC13566558; doi:10.3390/jcm15176556)
Supplement: Supplementary file 1 [file jcm-15-06556-s001.zip › Supplementary Table S1.pdf]

Supplementary Table S1. Complete Database-Specific Literature Search Strategies

Search framework. The final search was conducted on 28 February 2026. Eligible publications were restricted to the period from 1 January 2016 through 31 January 2026 and to the English language. The search combined controlled vocabulary, where available, with database-specific free-text terms for prior corneal refractive surgery and intraocular lens implantation. DOI availability, peer-review status, and article type were assessed during screening.

Database	Complete search strategy and limits
PubMed/MEDLINE	Search strategy:<br>(<br>"Refractive Surgical Procedures"[Mesh] OR "Keratomileusis, Laser In Situ"[Mesh] OR "Photorefractive Keratectomy"[Mesh] OR "Radial Keratotomy"[Mesh] OR "laser vision correction"[Title/Abstract] OR "corneal refractive surgery"[Title/Abstract] OR "refractive surgery"[Title/Abstract] OR LASIK[Title/Abstract] OR "laser-assisted in situ keratomileusis"[Title/Abstract] OR PRK[Title/Abstract] OR "photorefractive keratectomy"[Title/Abstract] OR SMILE[Title/Abstract] OR "small incision lenticule extraction"[Title/Abstract] OR "small-incision lenticule extraction"[Title/Abstract] OR "radial keratotomy"[Title/Abstract] OR "post-refractive"[Title/Abstract] OR "post refractive"[Title/Abstract] OR "post-LASIK"[Title/Abstract] OR "post LASIK"[Title/Abstract] OR "post-PRK"[Title/Abstract] OR "post PRK"[Title/Abstract] OR "post-SMILE"[Title/Abstract] OR "post SMILE"[Title/Abstract] OR "post-RK"[Title/Abstract] OR "post RK"[Title/Abstract] OR "myopic ablation"[Title/Abstract] OR "hyperopic ablation"[Title/Abstract]<br>)<br>AND<br>(<br>"Lenses, Intraocular"[Mesh] OR "Lens Implantation, Intraocular"[Mesh] OR "Cataract Extraction"[Mesh] OR "intraocular lens"[Title/Abstract] OR "intraocular lenses"[Title/Abstract] OR IOL[Title/Abstract] OR IOLs[Title/Abstract] OR "lens implantation"[Title/Abstract] OR "cataract surgery"[Title/Abstract] OR "premium IOL"[Title/Abstract] OR "premium intraocular lens"[Title/Abstract] OR "presbyopia-correcting IOL"[Title/Abstract] OR "presbyopia correcting IOL"[Title/Abstract] OR "presbyopia-correcting intraocular lens"[Title/Abstract] OR "presbyopia correcting intraocular lens"[Title/Abstract] OR multifocal[Title/Abstract] OR trifocal[Title/Abstract] OR quadrifocal[Title/Abstract] OR "extended depth of focus"[Title/Abstract] OR "extended range of vision"[Title/Abstract] OR EDOF[Title/Abstract] OR toric[Title/Abstract] OR accommodating[Title/Abstract] OR "wavefront-shaping"[Title/Abstract] OR "wavefront shaping"[Title/Abstract] OR "light-adjustable lens"[Title/Abstract] OR "light adjustable lens"[Title/Abstract] OR "small-aperture lens"[Title/Abstract] OR "small aperture lens"[Title/Abstract] OR "enhanced monofocal"[Title/Abstract]<br>)<br>AND ("2016/01/01"[Date - Publication] : "2026/01/31"[Date - Publication])<br>AND English[Language]
**Embase**	Search strategy:<br>('refractive surgery'/exp OR 'laser in situ keratomileusis'/exp OR 'photorefractive keratectomy'/exp OR 'radial keratotomy'/exp OR 'laser vision correction':ti,ab,kw OR 'corneal refractive surgery':ti,ab,kw OR 'refractive surgery':ti,ab,kw OR lasik:ti,ab,kw OR 'laser-assisted in situ keratomileusis':ti,ab,kw OR prk:ti,ab,kw OR 'photorefractive keratectomy':ti,ab,kw OR smile:ti,ab,kw OR 'small incision lenticule extraction':ti,ab,kw OR 'small-incision lenticule extraction':ti,ab,kw OR 'radial keratotomy':ti,ab,kw OR 'post-refractive':ti,ab,kw OR 'post refractive':ti,ab,kw OR 'post-lasik':ti,ab,kw OR 'post lasik':ti,ab,kw OR 'post-prk':ti,ab,kw OR 'post prk':ti,ab,kw OR 'post-smile':ti,ab,kw OR 'post smile':ti,ab,kw OR 'post-rk':ti,ab,kw OR 'post rk':ti,ab,kw OR 'myopic ablation':ti,ab,kw OR 'hyperopic ablation':ti,ab,kw)<br>AND<br>('intraocular lens'/exp OR 'intraocular lens implantation'/exp OR 'cataract extraction'/exp OR 'intraocular lens':ti,ab,kw OR 'intraocular lenses':ti,ab,kw OR iol:ti,ab,kw OR iols:ti,ab,kw OR 'lens implantation':ti,ab,kw OR 'cataract surgery':ti,ab,kw OR 'premium iol':ti,ab,kw OR 'premium intraocular lens':ti,ab,kw OR 'presbyopia-correcting iol':ti,ab,kw OR 'presbyopia correcting iol':ti,ab,kw OR 'presbyopia-correcting intraocular lens':ti,ab,kw OR 'presbyopia correcting intraocular lens':ti,ab,kw OR multifocal:ti,ab,kw OR trifocal:ti,ab,kw OR quadrifocal:ti,ab,kw OR 'extended depth of focus':ti,ab,kw OR 'extended range of vision':ti,ab,kw OR edof:ti,ab,kw OR toric:ti,ab,kw OR accommodating:ti,ab,kw OR 'wavefront-shaping':ti,ab,kw OR 'wavefront shaping':ti,ab,kw OR 'light-adjustable lens':ti,ab,kw OR 'light adjustable lens':ti,ab,kw OR 'small-aperture lens':ti,ab,kw OR 'small aperture lens':ti,ab,kw OR 'enhanced monofocal':ti,ab,kw)

Database	Complete search strategy and limits
	AND [english]/lim<br>AND [2016-2026]/py
**Web of Science Core Collection**	Search strategy:<br>TS=(<br>("laser vision correction" OR "corneal refractive surgery" OR "refractive surgery" OR LASIK OR "laser-assisted in situ keratomileusis" OR PRK OR "photorefractive keratectomy" OR SMILE OR "small incision lenticule extraction" OR "small-incision lenticule extraction" OR "radial keratotomy" OR "post-refractive" OR "post refractive" OR "post-LASIK" OR "post LASIK" OR "post-PRK" OR "post PRK" OR "post-SMILE" OR "post SMILE" OR "post-RK" OR "post RK" OR "myopic ablation" OR "hyperopic ablation")<br>AND<br>("intraocular lens" OR "intraocular lenses" OR IOL OR IOLs OR "lens implantation" OR "cataract surgery" OR "premium IOL" OR "premium intraocular lens" OR "presbyopia-correcting IOL" OR "presbyopia correcting IOL" OR "presbyopia-correcting intraocular lens" OR "presbyopia correcting intraocular lens" OR multifocal OR trifocal OR quadrifocal OR "extended depth of focus" OR "extended range of vision" OR EDOF OR toric OR accommodating OR "wavefront-shaping" OR "wavefront shaping" OR "light-adjustable lens" OR "light adjustable lens" OR "small-aperture lens" OR "small aperture lens" OR "enhanced monofocal")<br>)<br>Refined by: Languages = English<br>Timespan: 2016-2026
**Scopus**	Search strategy:<br>TITLE-ABS-KEY(<br>("laser vision correction" OR "corneal refractive surgery" OR "refractive surgery" OR LASIK OR "laser-assisted in situ keratomileusis" OR PRK OR "photorefractive keratectomy" OR SMILE OR "small incision lenticule extraction" OR "small-incision lenticule extraction" OR "radial keratotomy" OR "post-refractive" OR "post refractive" OR "post-LASIK" OR "post LASIK" OR "post-PRK" OR "post PRK" OR "post-SMILE" OR "post SMILE" OR "post-RK" OR "post RK" OR "myopic ablation" OR "hyperopic ablation")<br>AND<br>("intraocular lens" OR "intraocular lenses" OR IOL OR IOLs OR "lens implantation" OR "cataract surgery" OR "premium IOL" OR "premium intraocular lens" OR "presbyopia-correcting IOL" OR "presbyopia correcting IOL" OR "presbyopia-correcting intraocular lens" OR "presbyopia correcting intraocular lens" OR multifocal OR trifocal OR quadrifocal OR "extended depth of focus" OR "extended range of vision" OR EDOF OR toric OR accommodating OR "wavefront-shaping" OR "wavefront shaping" OR "light-adjustable lens" OR "light adjustable lens" OR "small-aperture lens" OR "small aperture lens" OR "enhanced monofocal")<br>)<br>AND PUBYEAR > 2015 AND PUBYEAR < 2027<br>AND (LIMIT-TO(LANGUAGE, "English"))
