# Supplementary material for: Premium Intraocular Lens Implantation After Laser Vision Correction: An Updated Narrative Review of Biometry, IOL Selection, Visual Outcomes, and Clinical Management
Source: J Clin Med. 2026 Aug 25;15(17):6556. doi: 10.3390/jcm15176556 (PMC13566558; doi:10.3390/jcm15176556)
Supplement: Supplementary file 1 [file jcm-15-06556-s001.zip › Supplementary Table S2.pdf]

Supplementary Table S2. Practical clinical decision-making framework for premium IOL implantation after laser vision correction

Decision point	Key clinical assessment	Practical decision-making approach	Caution / avoid when	Relevant references
Initial candidacy assessment	Type of previous LVC, refractive stability, corneal topography/tomography, optical zone, HOAs, and ocular comorbidity	Confirm stable refraction, regular corneal profile, adequate optical zone, and absence of visually significant comorbidity before selecting a premium IOL	Irregular cornea, unstable refraction, ectatic change, small optical zone, or reduced visual potential	[4,6,9,116,129]
Ocular surface preparation	Dry eye, tear-film instability, meibomian gland dysfunction, and repeatability of keratometry/biometry	Optimize the ocular surface before final biometry and repeat measurements when values are inconsistent	Severe untreated ocular surface disease, unreliable keratometry, or fluctuating vision	[24,49-51]
IOL power calculation	Type of prior refractive surgery, availability of historical data, posterior corneal power, total keratometry, and formula agreement	Use post-refractive formulas and compare multiple calculation methods; consider total keratometry, measured posterior corneal power, or intraoperative aberrometry when appropriate	Large disagreement between formulas or poor-quality measurements should prompt repeat assessment before surgery	[7-13]
IOL type selection	Visual needs, night-driving requirement, tolerance of halos/glare, corneal regularity, HOA burden, and residual astigmatism	Consider EDOF or enhanced monofocal designs in borderline corneal optics; reserve multifocal/trifocal IOLs for carefully selected eyes with regular corneas and realistic expectations	High HOAs, irregular astigmatism, small optical zone, strong night-vision demands, or low tolerance of dysphotopsia	[4,15,19,32,34,38,82-85]
Astigmatism planning	Magnitude and regularity of corneal astigmatism, axis stability, and agreement between measurement devices	Consider toric IOLs when astigmatism is regular and reproducible; confirm axis alignment and rotational stability postoperatively	Irregular astigmatism, unstable measurements, or poor agreement between devices	[4,9,73,129]

Decision point	Key clinical assessment	Practical decision-making approach	Caution / avoid when	Relevant references
Postoperative dissatisfaction	Residual refractive error, ocular surface disease, PCO, IOL tilt/decentration, and dysphotopsia	Treat reversible causes first, optimize ocular surface, correct stable residual refractive error when appropriate, and allow neuroadaptation when symptoms are tolerable	Avoid early surgical intervention unless symptoms are severe, persistent, and clearly lens-related	[21,52,85,112,117]
Persistent severe symptoms	Intolerable dysphotopsia, significant refractive surprise, confirmed lens-related cause, and failure of conservative measures	Consider corneal enhancement, piggyback IOL, or IOL exchange after careful counseling and stability assessment	IOL exchange should be considered cautiously after reversible causes have been addressed	[73,112,121,127]

Abbreviations: EDOF, extended depth of focus; HOA, higher-order aberration; IOL, intraocular lens; LVC, laser vision correction; PCO, posterior capsule opacification; RK, radial keratotomy.
